# Visual parameter optimisation for biomedical image processing

**DOI:** 10.1186/1471-2105-16-S11-S9

**Published:** 2015-08-13

**Authors:** AJ Pretorius, Y Zhou, RA Ruddle

**Affiliations:** 1 School of Computing, University of Leeds, Leeds, UK; 2 School of Computing, Engineering and Physical Sciences, University of Central Lancashire, Preston, UK

**Keywords:** visualisation, parameter optimisation, image analysis, image processing, biology, biomedicine, histology, design study

## Abstract

**Background:**

Biomedical image processing methods require users to optimise input parameters to ensure high-quality output. This presents two challenges. First, it is difficult to optimise multiple input parameters for multiple input images. Second, it is difficult to achieve an understanding of underlying algorithms, in particular, relationships between input and output.

**Results:**

We present a visualisation method that transforms users' ability to understand algorithm behaviour by integrating input and output, and by supporting exploration of their relationships. We discuss its application to a colour deconvolution technique for stained histology images and show how it enabled a domain expert to identify suitable parameter values for the deconvolution of two types of images, and metrics to quantify deconvolution performance. It also enabled a breakthrough in understanding by invalidating an underlying assumption about the algorithm.

**Conclusions:**

The visualisation method presented here provides analysis capability for multiple inputs and outputs in biomedical image processing that is not supported by previous analysis software. The analysis supported by our method is not feasible with conventional trial-and-error approaches.

## Background

Biomedical image processing is fundamental to many biological research methods [1]. These algorithms take parameter values and images as input, and produce annotated images and quantitative measures as output. Because they are sensitive to parameter values, imaging artefacts, and factors like tissue type, it is difficult to find robust parameter values that ensure high-quality output. Consequently, user judgment is an integral part of the optimisation process.

Optimisation problems may be classified in different ways, including the scale of parameter and output space. For the class of problem we consider, users deal with 2-7 input parameters and 2-7 output measures. Users also want to review image-based output for up to five images. We obtained these numbers by consulting domain experts and by observing users. They also correspond to observations in previous work [2-5]. There are problem classes with more parameters, but they are beyond the scope of this paper.

In this section we first review existing approaches for parameter optimisation. We then identify two important challenges (multiple inputs and outputs, and supporting understanding) and show that they are not addressed by this work. In further sections we describe a novel visualisation method to address the challenges and discuss a case study where our approach was used.

### Visual parameter optimisation

The most prominent approach for parameter optimisation is parameter tweaking. This involves repeatedly adjusting parameter values and reviewing output. It is tedious and incurs time and quality costs [5,6]. Automated parameter optimisation methods also exist, but require specialised mathematical insight and do not allow subjective analysis of output [7,8].

To address the shortcomings of parameter tweaking and automation, a number of visualisation methods have been developed (for example, see [9]). We classify them as follows. First, guided navigation approaches rely on an objective function or a distance measure from an ideal output (ground truth). Some show neighbourhoods in parameter space to guide users to optimal values [2,4]. Others support systematic exploration of parameter space by combining modelling, simulation, and visualisation [6,10]. These methods require an understanding of complex mathematical concepts for interpretation, which users may find challenging. Also, objective functions and ground truths are not always available (for example, see [5,11]).

A second class of methods relies on interactive visual exploration and qualitative evaluation of output. This includes dynamic queries of distribution plots of input and output [12,13]. It is also possible to visualize the parameter search graph to let users revisit and refine existing outputs [14,15]. Other methods visually structure parameter space to support the identification of suitable values [3,16]. An alternative is to emphasise the characteristics of output space and to let users select the output that best suit their needs [17,18].

Third, parameter space is typically high-dimensional and standard multidimensional visualisation methods can be used. This includes: dimensional stacking, where data items are embedded in a hierarchy of nested scatterplots [19]; hierarchical clustering, similar to dimensional stacking, but nested dimensions are shown as a directed tree; scatterplot matrices, where a matrix of scatterplots shows all two-way combinations of dimensions [20]; and parallel coordinates, where every variable is represented by a parallel axis and data items by polylines that intersect the axes. Hierarchical clustering and parallel coordinates have been extended to embed one image per parameter combination [21].

Finally, there are methods specifically for considering parameters in conjunction with image-based output. "Open-box" methods are custom-developed for specific image processing algorithms [22-24]. As parameter values are changed, they show algorithm-specific intermediate measures and update an output image. In previous work, we presented a visualisation method to analyse input parameters and image-based output for arbitrary algorithms [5]. It shows a Cartesian sampling of parameter space as a tree with a node-link diagram. Users select smaller contiguous regions of sampled parameter space to view associated output images in a coordinated view. This was followed by a case study, where users were able to analyse larger parts of parameter space and achieved higher quality results compared to parameter tweaking [25].

### Challenges

We now describe two unaddressed challenges, identified by analysing the above case study, a review of related work, and discussions with domain experts (Broad Institute, Leeds, and TU Darmstadt). We omit guided navigation since objective functions and ground truths usually do not exist in our application domain.

#### Multiple inputs and outputs

Image processing algorithms require multiple input parameters to be set and users are usually interested in analysing the results of an algorithm on multiple input images (typically of the same class, for example, tissue type). Users also want to examine the multiple output images and multiple output measures generated during algorithm execution. Hence, users need to combine an objective analysis of parameters and measures with a subjective analysis of images.

In Table 1, we show the ways in which previous visualisation methods are deficient. All methods deal with multiple input parameters, though some only treat pairs [3,16]. However, none support the analysis of algorithms applied to multiple input images. Further, previous methods are not designed to support visual analysis of multiple output measures (some offer limited capabilities [3,12,13,22-25]). Standard multidimensional visualisation methods can visualise parameters and measures but generally do not cater for images (it is sometimes possible to show a single output image per parameter combination [21]).

**Table 1:** Summary of visual support for multiple inputs and outputs.

	Input parameters	Input images	Output measures	Output images
**Distribution plots **[12,13]	Supported	No	Supported	No
**Search graph **[14,15]	Supported (changes)	Single only	No	One per parameter set
**Structured parameter space **[3,16]	Pairs only	Single only [16]	Supported [3]	One per parameter set
**Structured output space **[17,18]	Supported (changes)	No	Single objective function	One per parameter set
**Dimensional stacking **[19]	Supported	No	Supported	No
**Hierarchical clustering **[21]	Supported	No	Supported	One per parameter set
**Scatterplot matrices **[20]	Supported	No	Supported	No
**Parallel coordinates **[21]	Supported	No	Supported	One per parameter set
**Parameters & images **[5,22-25]	Supported	Single only	Limited	One per parameter set

In sum, there is an unmet challenge to visually support analysis of multiple input parameters, input images, output measures, and output images.

#### Understanding

Helping users understand their image processing algorithms is an important requirement to achieve confidence and generalise findings. With confidence in optimal parameter values for input images of the same class (for example, a tissue type), users can automate the processing for large volumes of similar data. An understanding of underlying algorithms also lets users generalise their findings to process input images with different characteristics. Finally, there is a need to validate image processing algorithms and to identify errors, particularly in a research context.

In Table 2, we show that previous visualisation techniques provide incomplete support for understanding algorithms. A few were designed to support understanding of specific algorithms, but do not generalise beyond that [22-24]. There are also methods that emphasise relationships between input and output. For example, our previous work helped users discover implementation errors and a logic error in a segmentation algorithm [25], while another allows for relating similar or erroneous output to parameter values [21]. Some methods let users investigate different scenarios in terms of input parameters [3,12,13], support the parameter search process [14,15], or permit exploration of simulations in a goal-oriented manner [17]. Nonetheless, they are geared to finding suitable parameter values.

**Table 2:** Summary of visual support for algorithm understanding.

	Supported	Unsupported
**Distribution plots **[12,13]	Relations between parameters and measures	Analysis of images
**Search graph **[14,15]	Identification of single suitable output image; relations between single input image and input parameter values	Analysis of multiple images, input parameters, or output measures
**Structured parameter space **[3,16]	Identification of suitable output images; relations of pairs of input parameters and output images	Analysis of multiple images or output measures
**Structured output space **[17,18]	Relations between input parameters and output images	Analysis of input images or output measures
**Dimensional stacking **[19]	Relations between parameters and measures	Analysis of images
**Hierarchical clustering **[21]	Relations between parameters and measures; relations between input parameters and one output image per combination	Analysis of input images; support for multiple output images
**Scatterplot matrices **[20]	Relations between parameters and measures	Analysis of images
**Parallel coordinates **[21]	Relations between parameters and measures; relations between input parameters and one output image per combination	Analysis of input images; support for multiple output images
**Parameters & images **[5,22-25]	Primarily, relations between input parameters and one output image per combination	Limited support for analysis of output measures; analysis of multiple images

We conclude that the challenge of supporting understanding is unmet. For previous visualisation methods, understanding is a bonus and not a design objective.

## Methods

To address the above challenges, we developed a visualisation technique to optimise parameters for biomedical image processing algorithms and implemented it in a tool called Paramorama2 [26]. Our technique is novel because: it enables holistic analysis of numerical and image-based inputs and outputs, and it provides interactive capabilities to enable flexible exploration of relationships between inputs and outputs. This work is the result of 30 months of close collaboration between the authors and diverse domain experts. Although our approach extends our previous work [5,25], there are important differences in conceptual approach, visual design, and the analysis it supports. In this section, we describe our design decisions.

Our data is generated offline by taking a Cartesian sampling of parameter space. For each (real-valued) input parameter, a user-specified interval is sampled. For each input image, the algorithm is executed once for each unique combination of sampled parameter values. This generates multiple output measures and output images that are associated with a particular combination of input parameters and the set of input images. Output measures are descriptive metrics that capture information about the output. We refer to a unique combination of input parameters, input images, output measures, and output images as a data record.

### Multiple inputs and outputs

As shown below, we combine a tabular visualisation of input parameters and output measures with an image browser for input and output images. We also describe design alternatives that we considered.

#### Tabular visualisation

**Figure 1:**
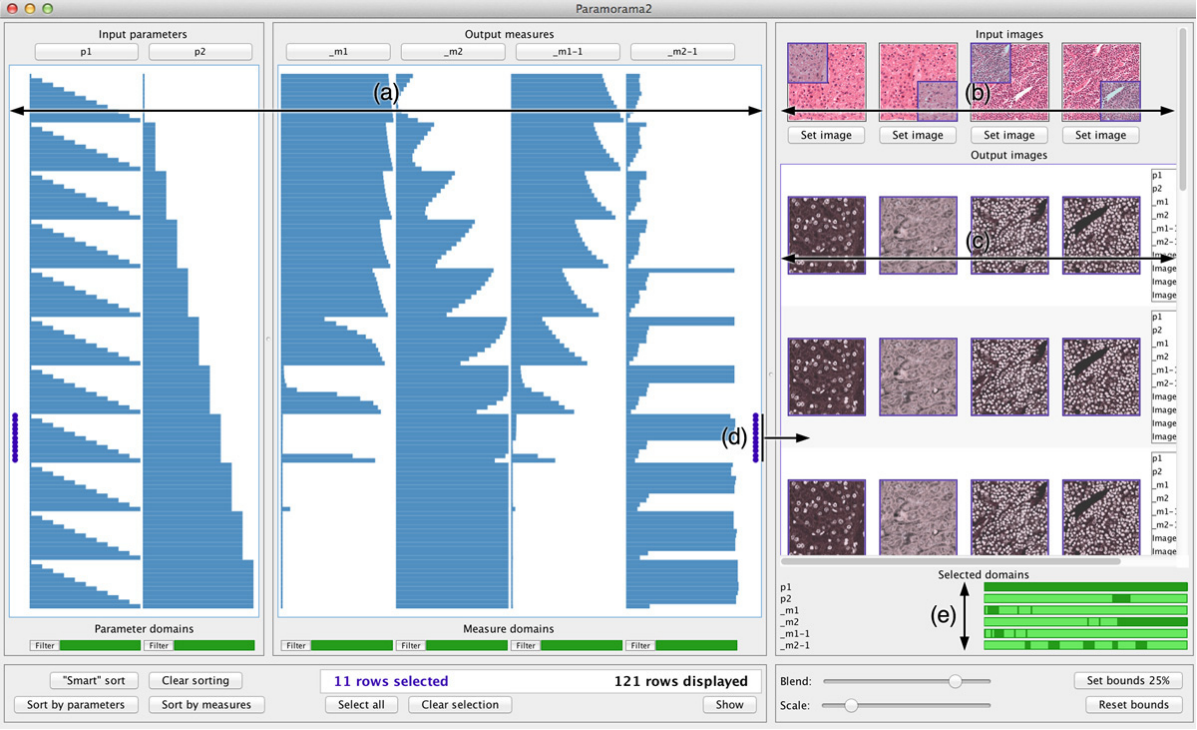
**Visual parameter optimisation for biomedical image processing**. (a) Every data record is represented by a row in a tabular visualisation, with columns for input parameters at the left and columns for output measures at the right. (b) Input images are shown at the top right of the image browser. (c) The image-based output produced for each input image is displayed below it in the image browser. (d) To view image-based output, users select rows in the tabular visualisation. The output images that are shown are the ones produced when the parameter values corresponding to the selected rows in the table are applied to the input images. (e) A list of selected parameters and measures is provided to show which parts of their domains the selected output images correspond to. The data shown here are from the case study and show results of a parameterised colour deconvolution technique applied to stained histology images of a liver section and lymphoma (a type of blood cancer).

We show the relationships between input parameters and output measures in a tabular visualisation (see Figure 1(a)). Columns at the left represent parameters and columns at the right represent measures. Each data record is represented by a row that spans across the columns. The value taken for a parameter or measure is encoded in the corresponding column. If the vertical space per row is more than four pixels, a bar chart encodes every column, otherwise a line chart is used. Although line charts do not prevent over-plotting, they are effective to let users discern high-level patterns when limited vertical space is available.

Our tables are similar to a table lens, which was developed primarily as a focus+context method [27]. Our objective, however, is to use the tabular representation to assist users in flexibly identifying and analysing relationships between parameters and measures. As we will show, we achieve this by extending our method with a number of interactive features.

#### Image browser

Our method has an image browser at the right of the user interface. It shows a horizontal list of input images at the top (see Figure 1(b)). When users select rows in the tabular visualisation, the corresponding output images are shown below the input images in a grid (Figure 1(c)). Column *i *shows the output images produced by applying the algorithm to the *i^th ^*input image. Each row represents a data record and the top-to-bottom order corresponds to the order of selected records in the tabular visualisation (Figure 1(d)).

By viewing the column of output images below each input image, users can compare output produced by different input parameter combinations for different input images. Each output image is blended with the input image to make comparisons easier (the amount of blending is user-specified). Users can also define a rectangular region of interest in each input image to view for the output images. This helps when there are particular regions that are known to be problematic for an algorithm (see Figure 1(b) and 1(c)). Users can also adjust output image magnification.

The primary mechanism to analyse relationships between input and output is interaction (see *Understanding*, below). As an additional aid, we provide a summary where horizontal strips represent the domains of parameters and measures (see Figure 1(e)). Dark regions indicate the values to which currently displayed images correspond.

#### Design alternatives

We also considered alternative visualisation methods. The most applicable highlight the structure of input or output space, but no existing ones integrate both (see Tables 1 and 2). Standard multidimensional visualisation methods were ruled out for the reasons below.

**Figure 2:**
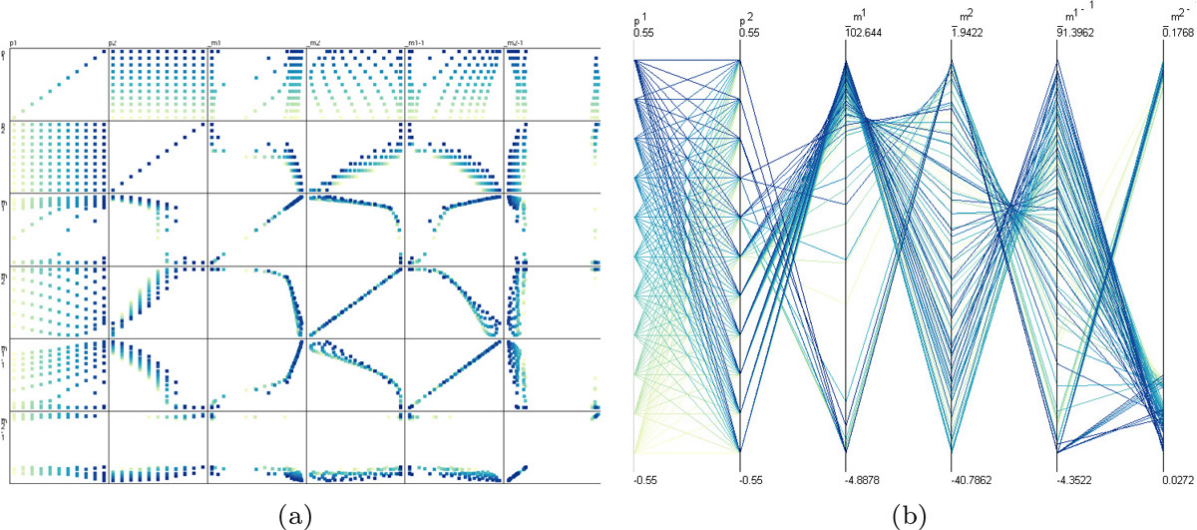
**Alternative visualisations of the data shown in Figure 1**. (a) A scatterplot matrix does not clearly show the multi-way correlations that appear as nested patterns in Figure 1(a). (b) Parallel coordinates require additional interaction, such as filtering, to identify these patterns. For both approaches, simple user interaction such as selection, is more complicated than with our method.

For dimensional stacking and hierarchical clustering, the real-estate requirements increase exponentially with the number of dimensions. Scatterplot matrices can visualise an arbitrary number of dimensions but, due to perceptual limitations, it is difficult to analyse relationships that span across more than two. For example, the multiway correlations that show up as nested patterns in Figure 1(a) cannot be easily discerned in Figure 2(a), which shows the same data. Our approach directly shows cyclical patterns in columns (for example, *m*_1_, *m*_2_, *m*_1 _− 1, and *m*_2 _− 1 in Figure 1). By contrast, parallel coordinates often mask such patterns when polylines overlay each other, requiring further interaction (see Figure 2(b)). To highlight cyclical patterns, we also considered spiral representations (for example, [28]). These require tuning an additional parameter to find a rotation interval and do not support multidimensional data. In fact, these alternatives all require far more effort for interacting with the data than our approach (see *Understanding*, below).

While developing our image browser we considered existing work for browsing photo libraries. Some, like PhotoFinder [29], show grids of sequentially ordered images. Others, like PhotoMesa [30], show the hard disk directory structure as a treemap. These methods were not designed to show relationships with and facilitate understudying of associated inputs and outputs.

### Understanding

Users need to discover and analyse relationships between input and output. Interaction is key to flexibly select data records and inspect associated images. For this, we combine column-based sorting, including automated sorting, with context-sensitive selection.

#### Column-based sorting

Users can interactively sort the rows in the tabular visualisation to identify relationships, such as correlations, that span across input parameter and output measure space. It is precisely this type of analysis that previous methods, including our own work, do not enable users to perform, instead focussing either on input or output (see Tables 1 and 2).

**Figure 3:**
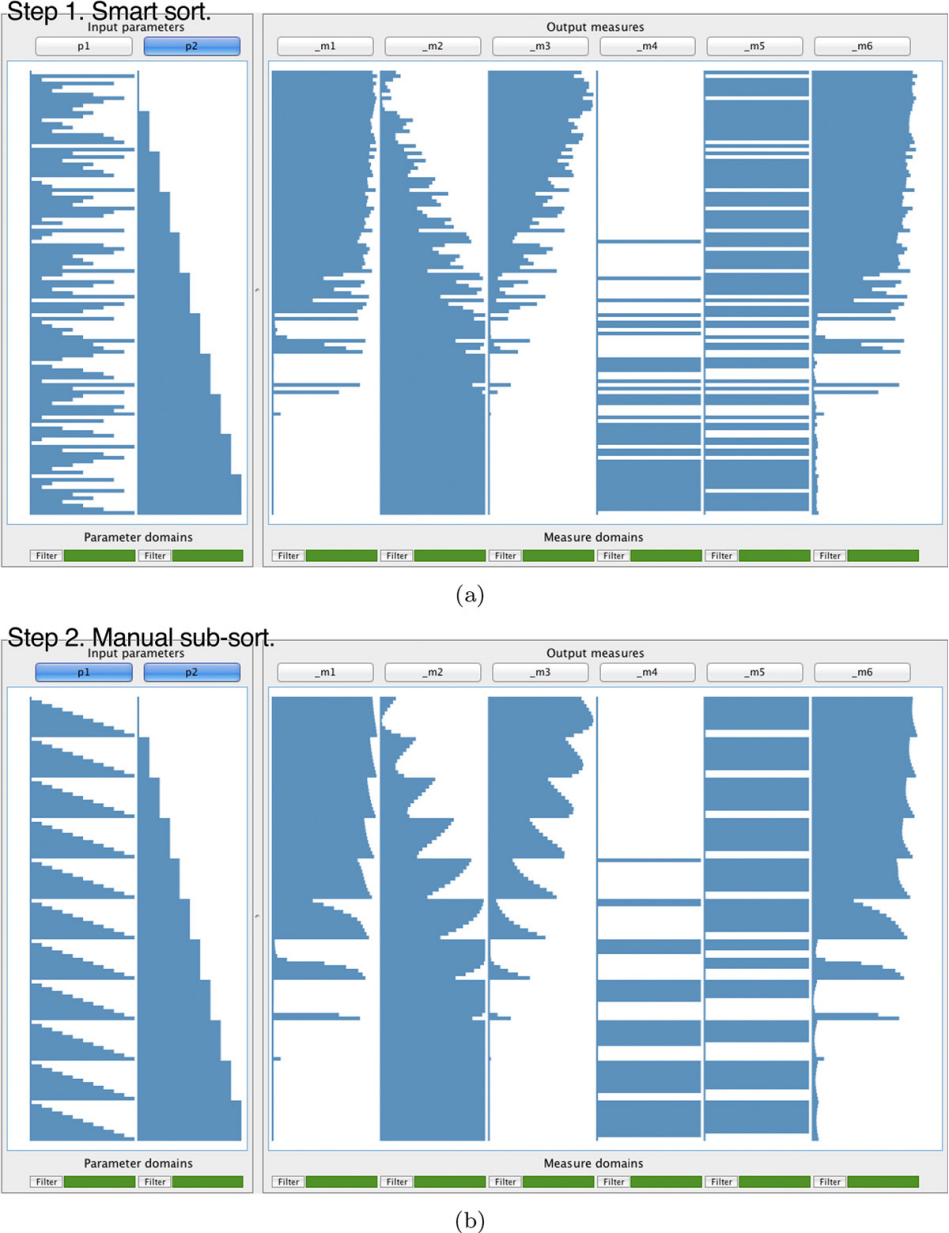
**Interactive sorting of input parameters of a colour deconvolution algorithm applied to a stained histology image of a liver section (see case study)**. (a) Applying "smart sorting" identifies input parameter *p*_2 _as the one with the highest aggregate correlation with variance of the output measures and sorts the rows of data according to values assumed for *p*_2_. This yields a step-like pattern with a bin for each unique value that *p*_2 _takes. Also, correlations between *p*_2 _and the output measures emerge, for example, *p*_2 _is directly correlated with *m*_2 _and inversely correlated with *m*_1_, *m*_3_, and *m*_6_. (b) Subsequent sorting on *p*_1 _reveals even more striking patterns. For example, in addition to the direct correlation with *p*_2_, *m*_2 _is also inversely correlated with *p*_1_.

When users click on a column header, data records are sorted by the values of the corresponding input parameter or output measure. In Figure 3(a), the data have been sorted by the second parameter (*p*_2_). A step-like pattern has emerged where records are grouped into a number of bins with the same value for parameter *p*_2_. It is possible to identify relationships between *p*_2 _and some of the output measures at the right. When multiple columns are selected, the order in which they were selected matters and all previously applied sortings are maintained. Figure 3(b) shows the result of sorting Figure 3(a) on *p*_1_. The records are only reordered within each of the bins of *p*_2 _to show a nested step-like pattern. Now, even more striking relationships with the output measures appear.

#### Automated sorting

During prototyping, we repeatedly observed users searching for the parameter that most highly correlates with output measures. We consequently implemented a simple automated sorting facility that we call "smart sorting". When users click on "Smart sort" (see Figure 1, lower left), our method computes the aggregate correlation of each input parameter and all output measures. The parameter with the highest correlation is identified and the data records are sorted by this parameter (Figure 3(a)).

#### Context-sensitive selection

**Figure 4:**
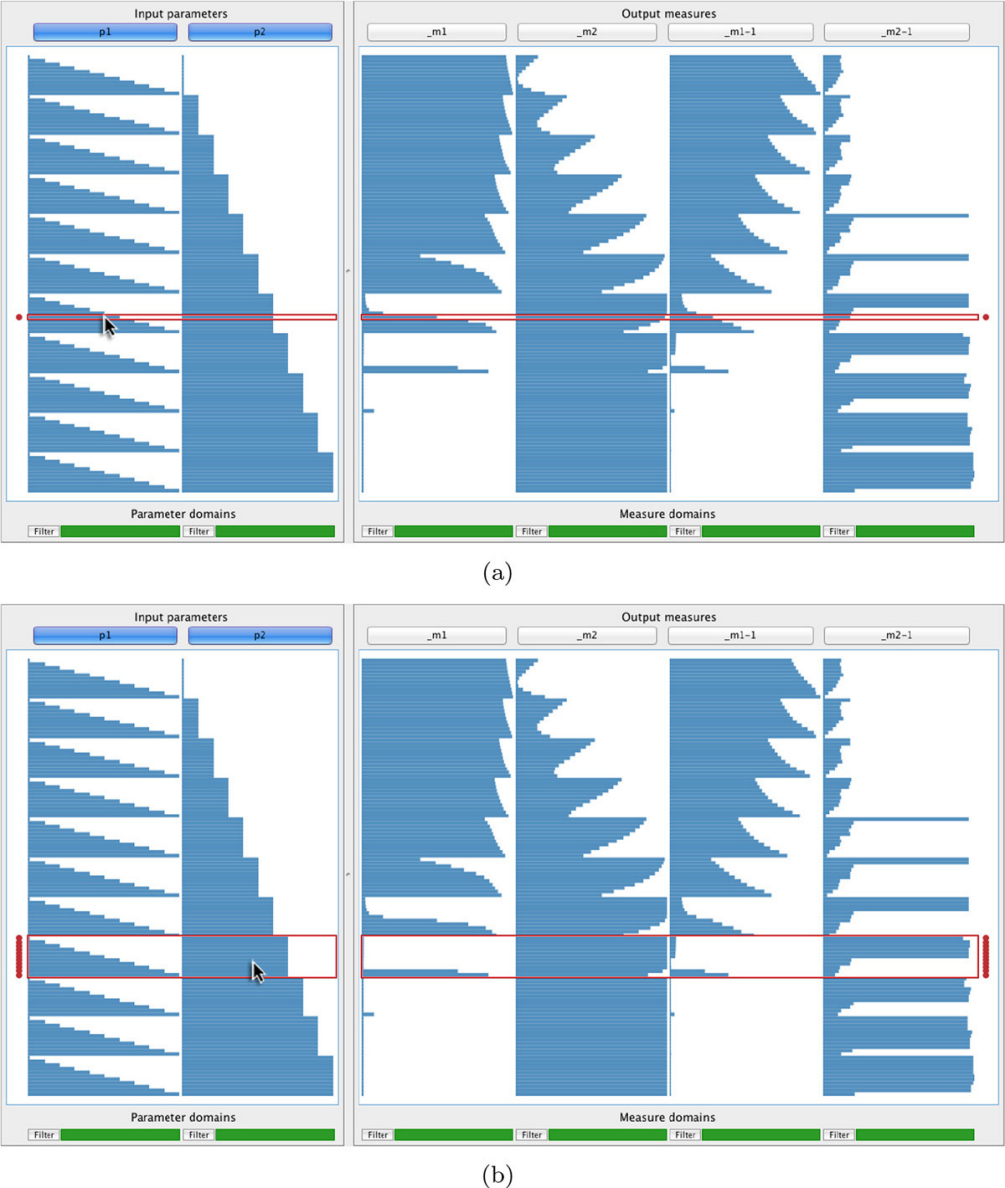
**Context-sensitive selection of the results in Figure 1 (see case study)**. When users move the cursor over the tabular visualisation, both the row and column that are intersected are considered. (a) If no immediately adjacent rows have identical values for the column under the cursor, a single row receives focus. (b) If adjacent rows do have the same value for the column under the cursor, they all receive focus. Clicking selects all rows that are in focus. Context-sensitive selection reduces effort to select multiple data records to display the corresponding output images in the image browser (see Figure 1(d)).

During prototyping, users found selection of individual data records too tedious to effectively analyse relationships between input and output. To address this, we developed context-sensitive selection. Suppose the cursor intersects row *r *and column *c*. In addition to highlighting row *r*, all directly adjacent rows with the same value for column *c *also receive focus. For example, compare the highlighted rows in Figure 4(a), where the cursor intersects column one, to Figure 4(a) where it intersects column two. Rows in focus are enclosed by a red frame and marked by two red disks in the margins. Clicking selects all rows in focus and marks each selected record with blue disks. Compound selections are made by multiple selections of this type.

Clicking on the button labelled "Show", below the tabular visualisation at the right, displays all images associated with selected data records in the image browser (Figure 1(d)). To provide more flexibility, users can rapidly filter records by clicking on "Filter" below any column and then specify an interval of interest. All records where the corresponding parameter or measure falls outside the interval are hidden. A strip below each column indicates which parts of its domain are currently displayed (see Figure 1).

There are situations where users want to look at output images associated with a single data record in real time. By holding the shift key, output images for the table row directly under the cursor are temporarily shown in the image browser. Images for a single record can be read and drawn at interactive speeds.

#### Design alternatives

We also investigated updating the image browser in real time as users select data records, or to use image caching. The former imposes a performance penalty for reading large numbers of images from disk, while the memory footprint of the latter limits scalability.

Column sorting combined with context-sensitive selection is an effective and efficient way to investigate meaningful subsets of data. We also considered "hard sorting" rows by column 1, then by column 2, and so on. This imposes a column-based hierarchy on the data and forces users to reorder columns to change the hierarchy. Instead, our approach lets users choose a column to sort on with one button click.

For automated sorting, it is possible to rank all input parameters on their individual correlations and to sort the data by all parameters, in this rank order. However, our users indicated that they prefer sorting by the single most significant parameter and our method therefore implements this approach. Providing automation as a "one-click" option, which can be visually verified and easily undone, alleviated fears about added complexity introduced by automated analysis.

## Results and Discussion

In this section, we describe applications of our method by providing an intuitive example and a case study. We also consider further biomedical applications, lessons learned, and opportunities for future work.

### Example: cell nuclei detection

**Figure 5:**
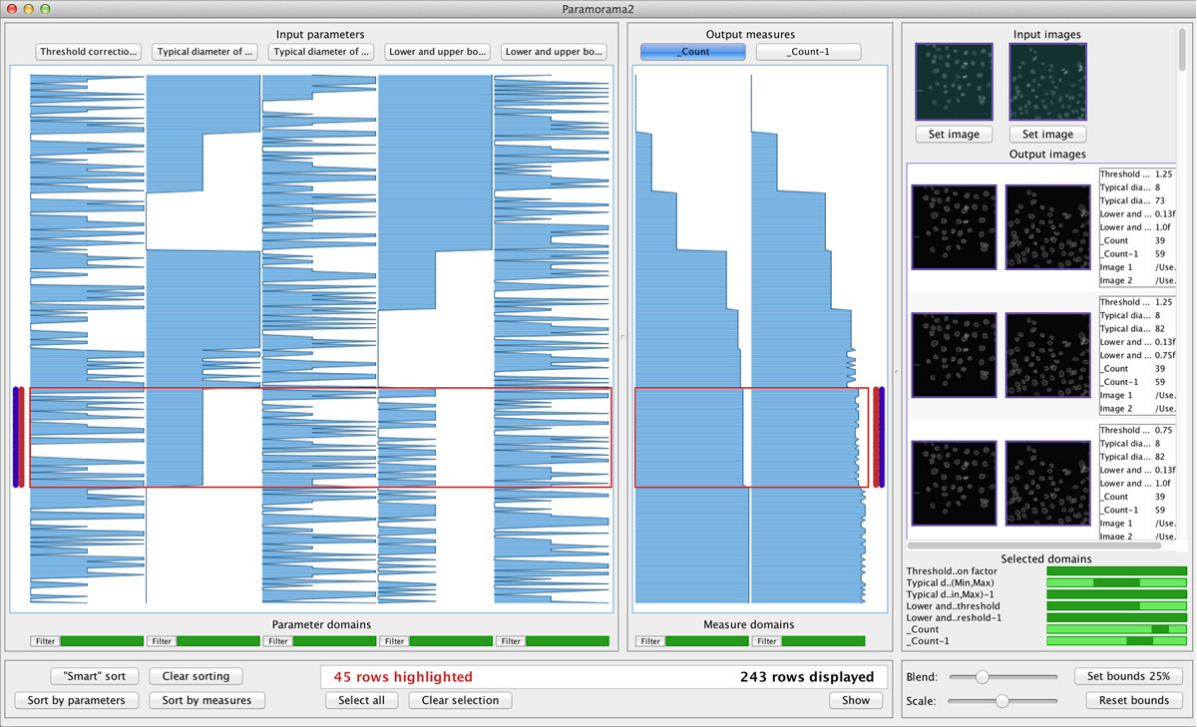
**The results of a nuclei detection algorithm on photomicrographs of human HT29 cells (colon cancer)**. The data have been sorted on the sixth column, which encodes the number of cells detected in the first input image. The highlighted rows indicate results where nuclei detection is correct and have been validated by also considering the output images at the far right. This reveals relationships with the values taken for the second and fourth input parameters (column two and four).

Cell nuclei detection is an important but challenging step in many high-throughput assays. As demonstrated in Additional file S1, Figure 5 shows the results of a cell nuclei detection algorithm for photomicrographs of human HT29 cells (colon cancer) that had been stained (Hoechst 33342) to highlight nuclei [31]. The algorithm has five input parameters that had been sampled three times each on meaningful intervals. For each combination of parameter values, the algorithm was run on two input images. For each parameter value combination, object counts for each image were captured as output measures and the outlines of detected nuclei were saved as output images.

The results have been sorted on column 6, the nuclei count for the first input image. This shows relationships with the second (*minimal nucleus diameter*) and fourth (*lower threshold*) parameters. By considering the values of the two parameters in combination with the output measures and images, it is straightforward to identify values for both that produce accurate nuclei detection (*nucleus diameter *takes its second value and *threshold *takes its first or second value). These results have been selected in Figure 5. A biomedical novice (first author) was able to identify these values in less than five minutes. The same task takes more than an hour with conventional parameter tweaking [5].

### Case study: colour deconvolution for histology

**Figure 6:**
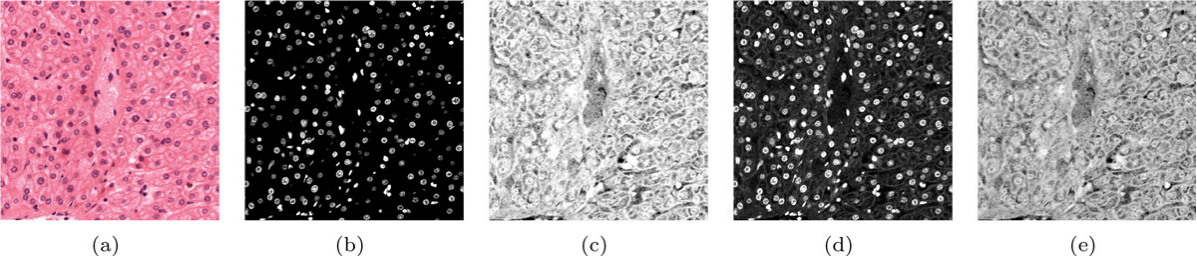
**Parameter optimisation for colour deconvolution of a histology image (see case study)**. (a) The original H&E stained image of a liver section. (b) Deconvolution result of the hematoxylin stain using default values. (c) Deconvolution result of the eosin stain using default values. (d) Deconvolution result of the hematoxylin stain using more optimal values found using our method. (e) Deconvolution result of the eosin stain using more optimal values. Note that the aim is not feature detection but accurate isolation of individual dyes, which have overlapping absorption rates in different sub-cellular structures. Results (d) and (e) reflect the absorption rates of the component stains more accurately than (b) and (c).

Histology is the study of tissue at a microscopic scale. Tissue is sectioned into micrometre-thin slices, fixed to glass slides, and stained with dyes that highlight different cellular compartments and structures. Hematoxylin and eosin dyes are often combined (H&E) to, respectively, colour cell nuclei blue and cytoplasm and connective tissue pink (see Figure 6(a)). However, different sub-cellular structures and proteins have overlapping absorption spectra, which makes it hard to differentiate contributions of individual dyes. Colour deconvolution is an image processing method that can extract individual dyes. This has important biological implications because it allows for quantitative tissue characterisation (see *Applications*, below).

For colour deconvolution, a suitable deconvolution matrix must be found. When applied to the original RGB image, this matrix splits it into several images, each representing the contribution of an individual dye. A biomedical image analysis expert (second author) had been investigating the assumed optimality of Ruifrok and Johnson's deconvolution matrix [32]. He had two objectives. *(O1) *Develop quality measures to quantify the performance of colour deconvolution. This requires an understanding of the behaviour of the deconvolution method. *(O2) *Find optimal values for two corrective parameters to optimise the deconvolution matrix. If non-zero, this would show that Ruifrok and Johnson's method is not optimal in all cases.

The expert had been working on the problem just short of a year when we got involved. The description below of how he achieved *O1 *and *O2 *with our method was obtained by a diary study and follow-up interview.

#### Preparation

The expert worked on two tissue types, liver and lymphoma (a type of blood cancer), which had been stained with H&E [33]. By using prior knowledge of the light absorption properties of tissue (the Beer-Lambert law [32]), the expert had developed two corrective parameters, *p*_1 _and *p*_2 _to apply to the deconvolution matrix and six candidate output measures to quantify the output quality.

The first parameter (*p*_1_) characterizes the haematoxylin stain in absorbing the red, green and blue components of the incident light. This parameter can be used to isolate cell nuclei in most cases. The second parameter (*p*_2_) characterises the eosin stain and can be used to isolate cytoplasm and connective tissue. Respectively, *m*_1_-*m*_3 _are the percentage of negative coefficients, mean value of negative coefficients, and standard deviation of negative coefficients corresponding to the first stain, while *m*_4_-*m*_6 _record the same results for the second stain. Detailed rationale of these parameters and measures are beyond the scope of this paper (involving optics and material light absorption properties), but an appreciation of the value of the insights gained with our method does not rely on a specialist understanding.

The expert was keen to find an alternative to parameter tweaking and immediately saw the potential of our method. He customised his software to sample the input parameters 11 times each on intervals identified based on domain knowledge. This yielded 121 unique combinations. Next, for each of the two input images, colour deconvolution was performed for each unique combination of sampled parameter values, and the corresponding data record was saved to disk. Input and output images measured 1, 000 × 1, 000 pixels.

#### Visual analysis

The expert's data is typical for our application domain. Each data record contained two input parameters, two input images, 12 output measures (six per input image), and four output images (two per input image). The expert started by analysing the deconvolution results for the liver section image (see Figure 6(a)).

*O1*. After loading the data, the expert applied smart sorting. This identified and sorted the data on *p*_2_, the parameter with the highest aggregate correlation with the variance of the output measures (see Figure 3(a)). The expert observed a number of patterns suggesting correlations between *p*_2 _and several of the output measures. Some appeared to be direct, while others appeared to be inverse relationships. The expert then sorted on *p*_1_. He was surprised by the result shown in Figure 3(b), where clear correlations between the input parameters and the output measures are evident. Before using our method, he had only seen single results (output image + quality scores) and had not been able to piece together the relationships shown here.

The expert now analysed these relationships (see Figure 3(b)). First, as *m*_4 _and *m*_5 _only took one of two values, they did not appear to carry sufficient information and the expert discarded them, reducing the complexity of the subsequent investigation. Next, the expert noticed that *m*_2 _and *m*_3 _appeared to be inversions of each other: when *m*_2 _increases, *m*_3 _decreases and vice versa. Finally, output measures *m*_1 _and *m*_6 _also closely resemble each other.

The expert concluded that *m*_1 _and *m*_2 _are sufficient to analyse the quality of results. The overview and analysis provided by our method enabled him to understand the behaviour shown by the output measures and, consequently, he was confident in this selection. He confirmed his choice by performing a similar analysis of the results of colour deconvolution on the lymphoma image. Figure 1 shows our method with the results of deconvolution applied to both input images. The two input parameters *p*_1 _and *p*_2 _are at the left of the tabular visualisation. At the right is the reduced set of output measures, *m*_1 _and *m*_2 _for the liver images and *m*_1−1 _and *m*_1−2 _for lymphoma. In this way, the expert was able to address *O1*.

*O2*. The results in Figure 1(a) show a direct relationship between the value assumed for parameter *p*_1 _and output measures *m*_1 _and *m*_1−1_. Based on the assumed optimality of Ruifrok and Johnson's deconvolution matrix [32], the expert expected to find high-quality output when *m*_1 _and *m*_1−1 _approach zero. Also, the parameters *p*_1 _and *p*_2 _represent deviations from the original deconvolution row vectors, where their 6*^th ^*value represents no change. Since the default deconvolution matrix was derived from numerous empirical experiments, the expert wanted to veer away from it as little as possible to achieve improved quality output. Having established *p*_2 _as the parameter most closely correlated with variation of the output measures, the expert decided to first review those data records closest to where *p*_2 _takes its 6*^th ^*value and where *m*_1 _and *m*_1-1 _approach zero. Figure 4(b) shows how he selected these records using context-sensitive selection.

The expert next reviewed the output images in the image browser. Because deconvolution splits each input image into two output images (one for hematoxylin, one for eosin), there are four columns of images in Figure 1. The first two columns correspond to the output for the liver section input image while the last two correspond to lymphoma. By reviewing these images, and cross-referencing parameters and measures, the expert identified a combination of input parameter values where *p*_2 _≠ 0 that yielded higher quality results for both input images than the original deconvolution matrix proposed in [32]. This enabled the expert to address *O2 *and show that the Ruifrok and Johnson deconvolution matrix is not always optimal.

#### Reflection

We followed up by conducting an unstructured debriefing interview. From this and our analysis of the case study, we conclude the following. First, with our approach the expert was able to effectively and efficiently address his research objectives. In particular, by addressing *O2*, our technique led him to a breakthrough in understanding. By discovering that an underlying assumption about the deconvolution algorithm he considered is invalid, he showed that Ruifrok and Johnson's deconvolution matrix is not optimal for all cases. Figure 6 illustrates this for the liver section.

Second, the total analysis time for both data sets was roughly 20 minutes. In contrast, the expert estimated that an attempt at a similar analysis using his conventional approach (parameter tweaking) would have required several days.

Third, the expert noted that despite previously focusing on *O1 *and *O2 *for nearly a year, he had little confidence in the results obtained with his conventional methods. By contrast, he was very confident of the results achieved with our method. In fact, based on his experience, he held a strong conviction that the rigour of analysis that our technique supports is practically unfeasible using his conventional approach.

### Applications

By applying the above algorithm to stained histology sections of engineered articular cartilage, scientists at the University of Leeds have found a direct correlation between stain intensity, which isolates an extracellular matrix, and the compressive strength of the cartilage. Cartilage repair with engineered tissue is an important new regenerative therapy for ageing populations and this approach offers a novel method for quantifying a key property using histology sections that are already routinely taken for subjective inspection.

Research into regenerative treatment also requires accurate models of, for example, spinal biomechanics. For this, biologists at Leeds are using the above algorithm to investigate if the distribution of stains obtained by deconvolution can be used to derive models of the structural properties of intervertebral disks.

Finally, image processing is a fundamental part of high-content screening workflows. Effective and efficient optimisation of these algorithms dramatically reduces the associated time and quality costs (for example, see *Example: cell nuclei detection*, above).

### Lessons learned

The parameter visualisation method described in this paper treats multiple input parameters, input images, output measures, and output images as first-class citizens for the first time. It results from an evolution in our understanding of the problem space. This is mirrored in the progression from our initial work that focuses only on input parameters and output for a single input image [5], to a limited and makeshift treatment of measures [25], to the work presented here. The long-term collaboration between us (first and third author) and diverse domain experts (like the second author) has been absolutely essential for this.

During this time, our collaborators' understanding and expectations of the role of visualisation also changed. Our joint work has convinced them that interactive visualisation is an important analysis paradigm. As our case study shows, visualisation enables them to address their problems in new and more effective ways.

By focusing on the problem, and not intrinsically on technical novelty, we were able to achieve a step-change for users. Due to the gap in previous parameter visualisation approaches, which are either parameter- or output-centric, they were limited in the types of analysis they could perform. Although our approach is partly based on existing methods, it combines these in a novel way to bridge this gap. By documenting the problem space and the design space, we argue that others will also be able to benefit from this work. This echoes calls for design studies by other authors [34,35].

### Future work

As we show in our case study, our approach increases effectiveness, efficiency and confidence in our application domain, where it is currently very challenging to analyse and understand relationships between multiple inputs and multiple outputs. Our approach also has limitations, however. The tabular visualisation was not designed to deal with over-plotting and, in practice, is limited to a maximum of about 7 parameters and a few thousand unique parameter value combinations. This deals with a class of problem that our target users typically face, but will not address all applications of parameter optimisation. For example, population models can contain hundreds of parameters [36].

Despite over-plotting, we have successfully analysed just over 17,000 data records of browsing behaviour from an unrelated usability study, where a total of nine parameters and measures were investigated and where sensitivity plots were viewed in the image browser. This suggests that our approach could scale to larger data sets than designed for and that it has potential for problems outside biomedical image processing.

Still, our approach requires sample sizes to be chosen in accordance with the number of data records to visualise. We see two ways to cater for scenarios that require greater scalability. First, a brute-force approach is to visualise more samples by using larger displays such as powerwalls, by letting visualisations scroll, or by implementing focus + context techniques (for example, [37]). A second approach is computational steering, where visualisation is integrated into a larger iterative cycle aimed at specifying and resampling regions of parameter space on the fly (for example, see [38]). We can, for instance, envision our visualisation interface integrated into an image processing framework like CellProfiler [39]. More work is required to investigate these possible approaches.

Another challenge is scaling to very large numbers of input and output images. Discussions with experts revealed that they would like to follow up on analyses like our case study with larger-scale validation exercises that involve hundreds or thousands of input images and their corresponding output. This would be valuable to validate the robustness of a set of parameter values. Here the emphasis shifts from dealing with the complexity of parameter space to also dealing with the complexity of very large collections of image-based input and output. There are currently no methods that enable users to interactively analyse the output generated for very large numbers of input images and this is an important open challenge for future research.

## Conclusions

We presented a visualisation technique for parameter optimisation of biomedical image processing algorithms. It addresses two challenges: dealing with multiple inputs and outputs (parameters, measures, and images) and enabling understanding of underlying algorithms. To show this, we provided a case study where a biomedical image processing expert used our method for colour deconvolution of histology images.

## Competing interests

The authors declare that they have no competing interests.

## Authors' contributions

AJP carried out visualisation design and implementation, conducted parts of the case study, and drafted the manuscript. YZ provided expert input for visualisation design, carried out and helped to draft the case study. RAR helped with visualisation design and to draft the manuscript. All authors read and approved the final manuscript.

## Supplementary Material

Additional File 1 Click here for file

## Declarations

Funding for publication of this article was provided by WELMEC, a Centre of Excellence in Medical Engineering funded by the Wellcome Trust and EPSRC (WT 088908/Z/09/Z).

This article has been published as part of *BMC Bioinformatics *Volume 16 Supplement 11, 2015: Proceedings of the 5th Symposium on Biological Data Visualization: Part 1. The full contents of the supplement are available online at http://www.biomedcentral.com/bmcbioinformatics/supplements/16/S11
